# Travel barriers, unemployment, and external fixation predict loss to follow-up after surgical management of lower extremity fractures in Dar es Salaam, Tanzania

**DOI:** 10.1097/OI9.0000000000000061

**Published:** 2020-03-03

**Authors:** Joseph T. Patterson, Patrick D. Albright, J. Hunter Jackson, Edmund N. Eliezer, Billy T. Haonga, Saam Morshed, David W. Shearer

**Affiliations:** a Institute for Global Orthopaedic Trauma, University of California San Francisco, San Francisco, CA; b Muhimbili Orthopaedic Institute, Dar es Salaam, Tanzania.

**Keywords:** clinical trial, femur, loss to follow-up, low-income country, orthopaedic trauma, secondary analysis, Tanzania, tibia

## Abstract

**Objective:**

Predict loss to follow-up in prospective clinical investigations of lower extremity fracture surgery.

**Design:**

Secondary analysis of 2 prospective clinical trials.

**Setting:**

National public orthopaedic and neurologic trauma tertiary referral hospital in Dar es Salaam, Tanzania, a low-income country in sub-Saharan Africa.

**Patients/Participants:**

Three hundred twenty-nine femoral shaft and 240 open tibial shaft fracture patients prospectively enrolled in prospective controlled trials of surgical fracture management by external fixation, plating, or intramedullary nailing between June 2015 and March 2017.

**Intervention:**

Telephone contact for failure to attend scheduled 1-year clinic visit.

**Main Outcome Measurements:**

Ascertainment of primary trial outcome at 1-year from surgery; post-hoc telephone questionnaire for reasons patient did not attend the 1-year clinic visit.

**Results:**

One hundred twenty-seven femur fracture (39%) and 68 open tibia fracture (28%) patients did not attend the 1-year clinic visit. Telephone contact significantly improved ascertainment of the primary study outcome by 20% between 6-month and 1-year clinic visits to 82% and 92% respectively at study completion. Multivariable analysis associated unemployment (OR = 2.5 [1.7–3.9], *P* < .001), treatment with an external fixator (OR = 1.7 [1.0–2.8], *P* = .033), and each additional 20 km between residence and clinic (OR = 1.03 [1.00–1.06], *P* = .047] with clinic nonattendance. One hundred eight (55%) nonattending patients completed the telephone questionnaire, reporting travel distance to the hospital (49%), and travel costs to the hospital (46%) as the most prevalent reasons for nonattendance. Sixty-five percent of patients with open tibia fractures cited relocation after surgery as a contributing factor.

**Conclusions:**

Relocation during recovery, travel distance, travel cost, unemployment, and use of an external fixator are associated with loss to clinical follow-up in prospective investigations of femur and open tibia fracture surgery in this population. Telephone contact is an effective means to assess outcome.

## 1 Introduction

Patient follow-up is a necessary part of clinical research. Loss to follow-up (LTFU) undermines the validity of conclusions drawn from clinical studies.^[1]^ LTFU in randomized controlled trials varies across medical and surgical research with a median rate of 6% (interquartile range of 2%–14%).^[2]^

Orthopaedic trauma surgery may be particularly vulnerable to LTFU. Traumatic pelvic and extremity injuries that interfere with physical function also interfere with travel to follow-up appointments. Patients who sustain orthopaedic trauma tend to be young, employed, and often have social and financial responsibilities to dependents that compete with researchers’ requests to return to clinical research sites for follow-up evaluations.^[1,3]^ These barriers to follow-up are problematic for Level I and II prospective trials in orthopaedic trauma surgery, for which LTFU rates range from 6% to 28%.^[3,4]^ Simulated reanalysis of randomized trials suggests that LTFU greater than 15% to 25% decreases the probability of finding a real difference in outcome between surgical interventions in orthopaedic trials.^[5]^

Identifying patients at greater risk of nonattendance of follow-up may inform the design of successful clinical trials in orthopaedic trauma surgery. In high-income countries (HIC), tobacco use, high-risk alcohol use, male sex, residence distant from the study site, nonprivate insurance, and hip and pelvis fractures have been associated with a greater risk of LTFU.^[3,6,7]^ Randomized trials in HIC have achieved less than 7% LTFU through active patient communication and tracking focused on patients at risk for LTFU.^[4,7]^ However, mitigating LTFU is particularly challenging in low- and middle-income countries (LMICs): physical and economic barriers to follow-up are more prevalent while human, financial, and technological resources to facilitate follow-up may be limited.^[8]^ Few investigators have published strategies for limiting surgical LTFU in LMICs. Sparse data suggest that travel distance and medical conditions that may interfere with travel may be prognostic of LTFU in these settings.^[9,10]^

The purpose of this study was to identify predictors of LTFU in prospective clinical investigations of lower extremity fracture surgery in a low-income country. We combined 2 cohorts of patients previously enrolled in separate prospective surgical trials at 1 tertiary orthopaedic surgical center in Tanzania, a low-income country in sub-Saharan African and surveyed their reasons for not returning to clinic. We hypothesize that demographic, injury, and treatment characteristics are predictive of LTFU after lower extremity trauma surgery.

## 2 Methods

A secondary analysis was performed with samples of 2 surgical trials conducted at 1 tertiary orthopaedic center in Dar Es Salaam, Tanzania. The Femur study was a prospective, controlled investigation of surgical treatment of diaphyseal femur fractures by intramedullary nail, plating, or external fixation enrolling from July 2012 to July 2013.^[11]^ Exclusion criteria were pathologic fracture, prior surgery involving the affected femur, presentation ≥ 6 weeks from injury, active infection at the planned surgical site at recruitment, severe traumatic brain injury, severe burns, and unwillingness or anticipated inability to complete follow-up clinic visits. Patients with ipsilateral tibia fractures were not excluded. The Femur study was funded by grants from the Orthopaedic Research and Education Foundation and Orthopaedic Trauma Association. The Tibia study was a prospective, randomized, controlled study investigating differential reoperation rates following surgical treatment of open diaphyseal tibia fractures by intramedullary nail or external fixation enrolling from June 2015 to March 2017.^[12]^ Exclusion criteria were traumatic wounds that required a flap for closures, vascular injury requiring repair, ipsilateral femur fracture, contralateral femur or tibia fracture, pathologic fracture, prior lower limb deformity, severe traumatic brain injury (Glasgow Coma Scale < 12), spinal cord injury, severe burns (> 10% total body surface area, or > 5% total body surface area with full thickness or circumferential injury), or unwillingness or anticipated inability to complete follow-up clinic visits. The Tibia study was funded by grants from Wyss Medical Foundation and Doris Duke Charitable Foundation. The Tanzanian Femur Trial, Tanzania Open Tibia Study, and this re-analysis were approved by the research ethics committees of the Muhimbili Orthopaedic Institute, Muhimbili University of Health and Allied Sciences, and University of California San Francisco (UCSF) IRB #11-07644 and #14-14792, respectively. DWS and ENE were co-primary investigators for the Tanzania Femur Trial. SM and BTH were coprimary investigators for the Tanzania Open Tibia Study. All patients completed written informed consent prior to enrollment.

Demographic, socioeconomic, injury, and treatment characteristics were recorded at enrollment in both trials. Clinic evaluations specified per study protocols at 2-week, 6-week, 3-month, 6-month, and 1-year study follow-up visits ascertained a common primary outcome of reoperation at the fracture site. Between the 6-month and scheduled 1-year study visits, study coordinators contacted patients or their relatives by telephone to request that patients attend an in-clinic follow-up appointment at 1 year after their index surgery. The purpose of the 1-year study visit was to obtain radiographs and a clinical examination documenting the presence or absence of a reoperation or a complication meriting a reoperation, as determined by the adjudication committee of the respective study. Routine and study-specific follow-up care was provided free of charge in separate Saturday clinics created and staffed solely for the purposes of study follow-up. No additional monetary reward, transportation compensation, or other financial incentive was provided to patients for completion of follow-up visits.

For this secondary analysis, participants of both studies were pooled then separated into 2 cohorts: those who completed an in-person clinic visit at 1 year from the index surgery and those who did not (LTFU). A telephone survey was introduced as a study modification to counter and characterize LTFU. Study coordinators surveyed participants (or family if the participant was not directly available) in the LTFU cohort from each study using a telephone questionnaire assessing subjective reasons for nonattendance of the 1-year follow-up visit (Supplement). The questionnaire evolved from the Femur study to reflect more direct and accessible language in the Tibia study, and additional questions were posed to better understand patient relocation behavior and payment concerns. For this reanalysis, the questions from each study were mapped to 1 of 6 domains: travel distance, travel cost, fear of hospital payment, feeling well, work obligations, and other medical issues (Table S1, Supplement). The primary study outcome of reoperation was also assessed subjectively and remotely at this telephone contact. Study coordinators collected data electronically using REDCap (Research Electronic Data Capture, Vanderbilt University, Nashville, Tennessee) hosted at UCSF. Road-travel distance to the clinic site from place of residence was estimated in kilometers using Google Maps (Google, Mountain View, California).

Demographic, socioeconomic, injury, and treatment variables collected at enrollment were compared between studies as well as by LTFU from either study. χ^2^ tests were used for categorical data. Student *t* test and Wilcoxon rank-sum tests were used for parametric and nonparametric continuous data, respectively. While the variables collected in each study overlapped sufficiently to pool data, these variables were not selected during the design of either trial with a specific intent for this reanalysis. Multivariable regression of LTFU with baseline variables common to both studies as well as study identity was performed by backwards stepwise regression with model optimization using the Akaike Information Criterion to determine independent predictors of LTFU. Hosmer and Lemeshow goodness-of-fit and likelihood ratio postestimation tests validated the multivariable model selection. Analyses were performed using Stata statistical software (version 15.0, StataCorp, College Station, Texas).

## 3 Results

Five hundred sixty-nine participants were enrolled among the 2 studies (329 Femur, 240 Tibia). Patients were predominantly male (85.8%) with an average age of 32.0 ± 11.0 years. Most were employed, participated in ambulatory or light work for their employment, and sustained less severe injuries by OTA Fracture Classification (59.3% type A fractures; Table 1).^[13]^ 5.1% of femur patients and 8% of tibia patients reported an address within 5 km of the clinic. Telephone access at enrollment among femur patients was 91% and open tibia patients 98%. Additional details of the study populations have been previously reported.^[11,12]^

**Table 1:**
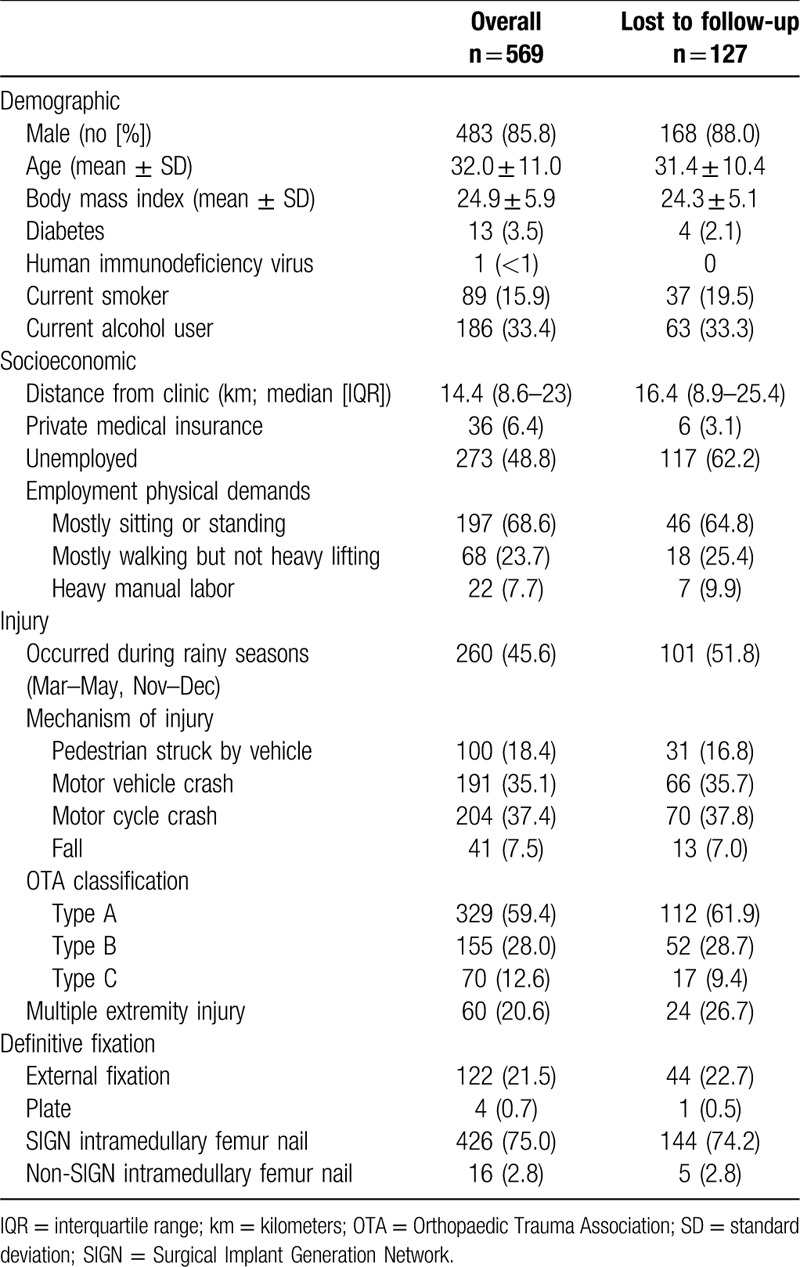
Baseline patient characteristics by completion of 1-year study follow-up in a clinic visit.

The overall loss to in-person clinic follow-up at 1 year was 195 (34.3%) patients. The rate of nonattendance of the 1-year study visit was significantly different between the femur study (n = 127, 38.4%) and the tibia study (n = 68, 28.3%; *P* = .013). The multivariable regression model considering baseline demographic, socioeconomic, injury, and treatment factors identified unemployment (odds ratio [OR] = 2.5 [1.6–3.8], *P* < .001), fracture stabilization with an external fixator (OR = 1.8 [1.1–2.9], *P* = .024), and incremental distance between the clinic and residential address at the time of injury (OR = 1.03 [1.00–1.06] per 20 km, *P* = .045) as independent predictors of LTFU (Table 2). Notably, sustaining a femoral shaft fracture versus an open tibial shaft fracture was not significantly associated with loss to follow-up after multivariable adjustment for baseline covariables (Table 3).

**Table 2:**
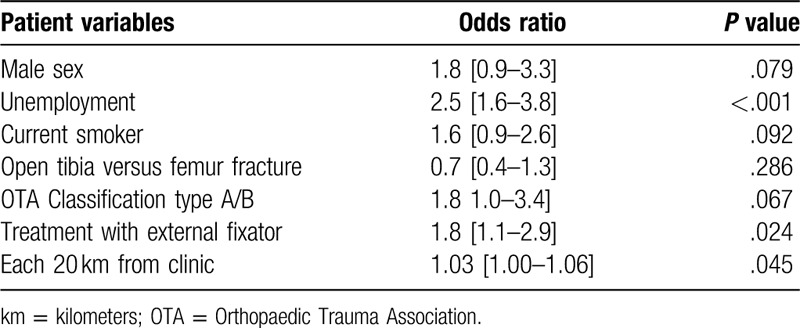
Multivariable analysis of patient characteristics associated with loss to follow-up.

**Table 3:**
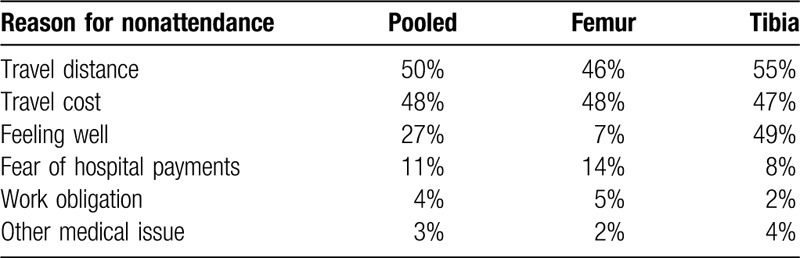
Telephone survey: patient agreement with reasons for nonattendance of 1-year clinic visit.

**Figure 1:**
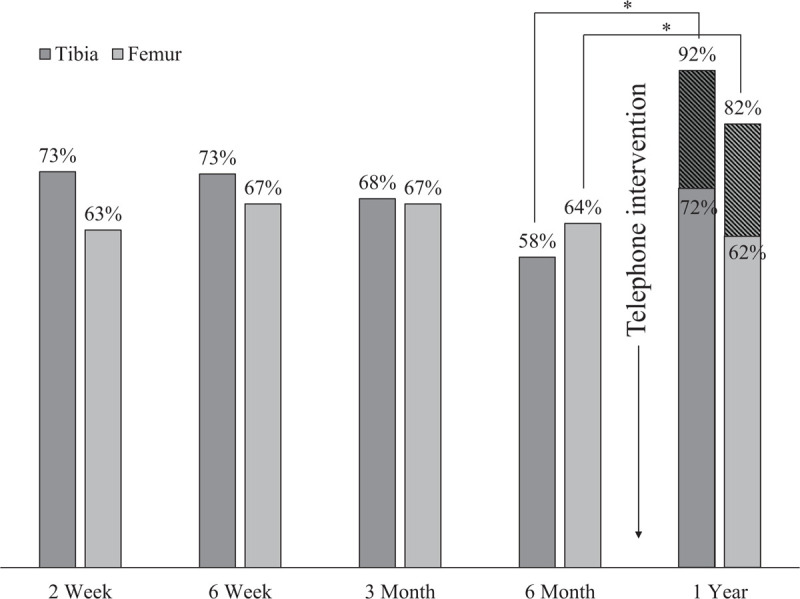
Telephone contact improves completion of primary study end-point. ∗Pearson χ^2^*P* value < .001. Diagonal lines indicate primary outcome was determined by telephone contact.

The telephone survey was completed by 108 individuals (68 femur, 49 tibia) lost to clinical follow-up at the 1-year study visit (55.3%). Figure 1 illustrates the loss to follow-up at each scheduled study visit, including the impact of this telephone contact between the 6 month and final study visits. Telephone contact ascertained the primary study outcome of reoperation in 20.7% of Femur study participants and 20.4% of Tibia study participants, improving study completion to 82.2% and 92.1%, respectively. Patients responding to the telephone questionnaire most commonly cited travel distance to the hospital (49%) and travel costs to the hospital (46%) as their reason for failing to complete in-clinic follow-up. The tibia cohort described feeling well (49%) as their reason for lack of follow-up while the femur cohort rarely cited this reason (7%). Additionally, the tibia cohort identified relocation (65%) as their reason for loss to follow-up. This question was not posed to the Femur study participants (Supplement) and was therefore excluded from pooled analysis.

## 4 Discussion

This study investigated subjective patient explanations as well as objective demographic, socioeconomic, injury, and treatment associations with failure to attend a 1-year, in-person study visit in 2 prospective clinical trials of lower extremity long-bone fracture care at a tertiary referral center in Tanzania. Thirty-four percent of trial participants did not attend their final, 1-year clinic visit. A telephone survey systematically distributed to these patients remotely assessed the primary study outcome, thereby improving study completion by 20% in each trial. Patients reported relocation during postoperative recovery, travel distance, travel cost, expected medical costs, and feeling well enough to require no additional clinical evaluation as subjective reasons for electing to not return for final follow-up. A multivariable secondary reanalysis also identified travel distance as well as unemployment and stabilization with an external fixator as independent predictors of loss to follow-up, but was not able to identify key patient-reported barriers to continued study participation.

Few studies have explored predictors of LTFU in LMICs. Prospective studies of surgical and medical interventions in sub-Saharan African LMICs report LTFU in the range of 16% to 43%.^[3,14–17]^ No other study, to our knowledge, has evaluated risk factors for LTFU after lower extremity trauma in large, prospective controlled trials in sub-Saharan Africa or quantitatively surveyed patients on reasons for nonattendance of follow-up. We identified barriers to follow-up in Dar es Salaam distinct from those in HIC orthopaedic trauma populations and comparable to other sub-Saharan African countries. Patients relocate during recovery and perceive this as a barrier to follow-up. We confirm that travel distance to clinic is a barrier to follow-up in Tanzania as reported in Ghana, Kenya, Malawi, and South Africa.^[10,14,16,17]^ While modifications to study design, case management, and behavioral interventions have proven effective in mitigating the barriers of travel distance in both LMICs and HIC,^[7,8,10,15,18]^ we demonstrate that unique strategies are needed to address unique barriers in a LMIC orthopaedic trauma population.

In HICs, the pooled rate of follow-up in prospective orthopaedic trauma research is 72% to 96%.^[3,11,16,17]^ The FLOW study, a multicenter study of differing fluid pressures and solutions used for debridement and irrigation for open extremity fractures performed in HIC in North America, Australia, Europe as well as India (a lower-middle income country at the time of the study) achieved 94% in-person or telephone follow-up without financial incentives through using predetermined strategies to limit LTFU.^[7,19]^ Through a similar secondary reanalysis, the FLOW Investigators identified age, male sex, current smoking, high-risk alcohol consumption, polytrauma, severe fracture grades, and private insurance as risk factors for LTFU—though patients were not queried as to their own reasons for attrition.^[7]^ That impressive rate is difficult to compare with the trials discussed herein. Virtually all subjects in our studies would have been excluded per the FLOW study protocol: Tanzanian patients rarely have fixed numeric addresses, 65% of tibia patients planned to relocate during recovery, and populations were sampled from distinctly different socioeconomic and geographical contexts (16 [Study Protocol and Supplementary Appendix]). The higher rate of LTFU in LMICs suggests that the population and environment in which these injuries occur may have a greater influence on return to clinic and study participation than the injury or treatment.

Travel is a barrier to care in sub-Saharan Africa. Limited density of surgical specialists may require patients to travel hundreds of kilometers. Patients often lack personal transportation; may have limited access to seasonally impractical shared transportation options; and may lack financial resources for their journeys. Fracture patients presenting to Dar es Salaam most frequently reported travel barriers (travel distance, travel cost, and relocation) as reasons for electing to not return. Less than 10% of patients in our studies reported a residence within a reasonable walking distance of 5 km of our clinic at the time of injury, and 65% relocated after injury. Our experience is consistent with reports across sub-Saharan Africa: 71% of ankle fracture patients in South Africa were obligated to travel a mean distance of 460 km.^[10]^ 42.% of Kenyan male circumcision patients lost to follow-up faced at least 8 hours of travel to reach the hospital.^[14]^ Young et al^[17]^ reported 42% loss to in-clinic follow-up after intramedullary nailing of femoral shaft fractures in Malawi. Through telephone interviews and outreach visits, they described anecdotal challenges with transportation infrastructure, seasonal rains, registration data, and patient access to telephones.^[17]^

Employment data was initially collected to ascertain return to work after injury. Incidentally, employment at the time of injury was independently associated with return for follow-up visits, a strong finding with a 98% response rate. Employment status may represent a proxy for socioeconomic status in both HICs and LMICs. However, the type of orthopaedic condition, occupation, and income may confound any relationship between employment and follow-up. Employment reportedly conferred a greater risk of LTFU in HIC hand surgery patients but was protective in spinal surgery patients,^[20,21]^ while income strata and specific forms of employment (unskilled clerical, sales, service, and labor occupations) were associated with LTFU in a Canadian lower extremity trauma population.^[22]^ The rate of unemployment at the time of injury among our Tanzanian lower extremity trauma patients was far greater than the municipal unemployment rate of 10% to 12% in Dar es Salaam for the study period,^[23]^ which may reflect the economic status of this population. A limitation of the present study is that we did not collect more robust metrics of socioeconomic status and employment data in both trials.

We did not establish a distinction between femur and tibia fractures with regard to follow-up after adjustment for other baseline factors. Neither the FLOW trial LTFU reanalysis nor any other previous LTFU analysis in high- or middle-income countries has compared follow-up rates by high versus low-middle income nations, or by fracture location including long bone (femur versus tibia) or long bone versus minor appendicular bone (scapula, clavicle, wrist, foot).^[7]^

External fixation versus intramedullary nailing of femur and open tibia fractures was independently associated with higher risk of loss to follow-up. We are not aware of a previous report of an orthopaedic implant associated with greater risk of failure to return to clinic. Because we did not ask patients directly about their attitudes toward each type of implant, we can only speculate as to the root of this finding. One possibility is that after implant removal, which was routine for external fixation, patients were more likely to “feel well” and not return for long-term follow-up visits. Patients with intramedullary nails, which were not routinely removed, were perhaps more likely to attribute residual symptoms to their retained intramedullary implant and therefore seek additional follow-up care.

There are limitations to this secondary reanalysis of 2 prospective trials. The covariables collected were not selected a priori for the purpose of investigating loss to follow-up. The multivariable model is thus limited by selection bias and clearly excludes key patient concerns identified in the survey. The pooled analysis is also limited to variables common to both studies, which omitted some socioeconomic information (education, household income, household members), surgeon training level, and time to treatment as collected in the Tibia study because the preceding Femur study did not gather these data. We did not anticipate patient relocation or the impact of transportation costs, and found no feasible means of objectively estimating transportation costs due to the variety of modes of transport used by our patients and limited use or retention of receipts for transportation charges. Few patients in this study self-reported HIV status, which is widely discrepant from Tanzanian national prevalence statistics as well as regional prevalence reported in femoral shaft fracture patients in Malawi.^[17]^ HIV is strongly associated with mortality and clinical trial attrition in this region.^[8,15]^ Nonetheless, these trials provide proof of concept that effective and rigorous prospective orthopaedic studies can be completed in LMICs with high follow-up rates. This study is strengthened by generalizability to orthopaedic trauma research in sub-Saharan East Africa as evidenced by similar sample demographics, injury mechanisms, and distance from treating center to patient residence as reported by a trauma referral center in a neighboring country.^[8,17]^

The findings of this investigation changed our research practice. At screening, telephone ownership is now mandatory for trial inclusion. Patients are excluded if study outcomes require in-person assessment and the participant plans to relocate distant to the great Dar area during convalescence from their injury. For outcomes, we developed and validated an outcome tool for femur fracture that does not require in-person administration to obviate the need for travel to in-person study visits.^[24]^ To offset travel barriers, we now compensate patients equally at study completion for travel distance and cost.

In conclusion, employment status, use of external fixator, and travel distance from clinic are associated with risk of loss to follow-up in lower extremity orthopaedic trauma patients in Dar es Salaam, Tanzania. Telephone contact, separate clinics for study-related care, no charge for study-related care, and explicit reminders of no-cost care appear to improve attendance of study follow-up visits. Relocation after injury, travel distance, travel cost, and perception of no medical need are barriers to convincing patients to return for follow-up. These findings provide opportunities to improve future trial design and consider behavioral interventions in orthopaedic trauma clinical research in this setting.

## Supplementary Material

Supplemental Digital Content

## Abbreviations

Abbreviations: OR = odds ratio; km = kilometer.

## References

[1] Zelle BA Bhandari M Sanchez AI Loss of follow-up in orthopaedic trauma: is 80% follow-up still acceptable? J Orthop Trauma. 2012;27:177–181. 10.1097/BOT.0b013e31825cf367 23449099

[2] Akl EA Briel M You JJ Potential impact on estimated treatment effects of information lost to follow-up in randomised controlled trials (LOST-IT): systematic review. BMJ. 2012;344:e2809. 22611167 10.1136/bmj.e2809

[3] Somerson JS Bartush KC Shroff JB Loss to follow-up in orthopaedic clinical trials: a systematic review. Int Orthop. 2016;40:2213–2219. 27142421 10.1007/s00264-016-3212-5

[4] Sprague S Leece P Bhandari M Limiting loss to follow-up in a multicenter randomized trial in orthopedic surgery. Control Clin Trials. 2003;24:719–725. 14662277 10.1016/j.cct.2003.08.012

[5] Zelle BA Panzica M Vogt MT Influence of workers’ compensation eligibility upon functional recovery 10 to 28 years after polytrauma. Am J Surg. 2005;190:30–36. 15972167 10.1016/j.amjsurg.2005.01.042

[6] Whiting PS Greenberg SE Thakore RV What factors influence follow-up in orthopedic trauma surgery? Arch Orthop Trauma Surg. 2015;135:321–327. 25617213 10.1007/s00402-015-2151-8

[7] Madden K Scott T McKay P Predicting and preventing loss to follow-up of adult trauma patients in randomized controlled trials: an example from the FLOW trial. J Bone Joint Surgery Am. 2017;99:1086–1092. 10.2106/JBJS.16.00900 PMC5490332 28678121

[8] Young S. Orthopaedic trauma surgery in low-income countries. Acta Orthopaedica. 2014;85 (SUPPL. 356):1–35. 10.3109/17453674.2014.937924 25052728

[9] Delamou A Delvaux T Utz B Factors associated with loss to follow-up in women undergoing repair for obstetric fistula in Guinea. Trop Med Int Health. 2015;20:1454–1461. 26250875 10.1111/tmi.12584

[10] Badenhorst DHS Van der Westhuizen CA Britz E Lost to follow-up: Challenges to conducting orthopaedic research in South Africa. S Afr Med J. 2018;108:917–921. 30645956 10.7196/SAMJ.2018.v108i11.13252

[11] Eliezer EN Haonga BT Morshed S Predictors of reoperation for adult femoral shaft fractures managed operatively in a Sub-Saharan country. J Bone Joint Surg Am. 2017;99:388–395. 28244909 10.2106/JBJS.16.00087

[12] Ibrahim J Liu M Yusi K Conducting a randomized controlled trial in Tanzania: Institute for Global Orthopaedics and Traumatology and the Muhimbili Orthopaedic Institute. J Orthop Trauma. 2018;32:S47–S51. 30247401 10.1097/BOT.0000000000001294

[13] Meinberg E Agel J Roberts C Fracture and Dislocation Classification Compendium—2018. J Orthop Trauma. 2018;32:S1–S170. 10.1097/BOT.0000000000001063 29256945

[14] Bailey RC Moses S Parker CB Male circumcision for HIV prevention in young men in Kisumu, Kenya: a randomised controlled trial. Lancet. 2007;369:643–656. 17321310 10.1016/S0140-6736(07)60312-2

[15] Geng EH Bangsberg DR Musinguzi N Understanding reasons for and outcomes of patients lost to follow-up in antiretroviral therapy programs in Africa through a sampling-based approach. J Acquir Immune Defic Syndr. 2010;53:405–411. 19745753 10.1097/QAI.0b013e3181b843f0 PMC3606953

[16] Teelken MA Stienstra Y Ellen DE Buruli ulcer: differences in treatment outcome between two centres in Ghana. Acta Trop. 2003;88:51–56. 12943977 10.1016/s0001-706x(03)00170-0

[17] Young S Banza LN Hallan G Complications after intramedullary nailing of femoral fractures in a low-income country. Acta Orthop. 2013;84:460–467. 24171678 10.3109/17453674.2013.850014 PMC3822130

[18] Casp AJ Wells J Holzgrefe R Evaluation of orthopedic trauma surgery follow-up and impact of a Routine Callback Program. Orthopedics. 2017;40:e312–e316. 28056157 10.3928/01477447-20161229-01

[19] Bhandari M Jeray KJ Petrisor BA. The FLOW Investigators A trial of wound irrigation in the initial management of open fracture wounds. N Engl J Med. 2015;373:2629–2641. 26448371 10.1056/NEJMoa1508502

[20] Ootes D Buijze GA Ring D. Predictors of missed appointments in prospective hand surgery research. Hand (N Y). 2012;7:177–180. 23730237 10.1007/s11552-012-9411-7 PMC3351508

[21] Sielatycki JA Parker SL Godil SS Do patient demographics and patient-reported outcomes predict 12-month loss to follow-up after spine surgery? Spine (Phila Pa 1976). 2015;40:1934–1940. 26595443 10.1097/BRS.0000000000001101

[22] Murnaghan ML Buckley RE. Lost but not forgotten: patients lost to follow-up in a trauma database. Can J Surg. 2002;45:191–195. 12067171 PMC3686949

[23] Tanzania National Bureau of Labour Statistics. Employment Estimates Brochure for Tanzania Mainland 2015-2017 [Internet]. [cited 2019 Aug 17]. Available at: https://www.nbs.go.tz/index.php/en/census-surveys/labour-statistics. Accessed August 17, 2019.

[24] Wu H-H Liu M Challa ST Development of Squat-and-Smile test as proxy for femoral shaft fracture-healing in patients in Dar es Salaam, Tanzania. J Bone Joint Surg Am. 2019;101:353–359. 30801374 10.2106/JBJS.18.00387

