# Supplementary material for: Travel barriers, unemployment, and external fixation predict loss to follow-up after surgical management of lower extremity fractures in Dar es Salaam, Tanzania
Source: OTA Int. 2020 Mar 3;3(1):e061. doi: 10.1097/OI9.0000000000000061 (PMC8081490; doi:10.1097/OI9.0000000000000061)
Supplement: Supplemental Digital Content [file oi9-3-e061-s001.docx]

Table S1. Risk of loss to one-year clinical follow-up by baseline patient characteristics.

| ***Demographic*** | **Relative Risk** | **p-value** |
| --- | --- | --- |
| Male (*no [%])* | 1.2 (0.8 - 1.7) | 0.291 |
| Age (mean ± SD) | - | 0.390 |
| Body mass index (mean ± SD) | - | 0.071 |
| Diabetes | 0.7 (0.3 - 1.6) | 0.343 |
| Human immunodeficiency virus | - | - |
| Current smoker | 1.3 (1.0 - 1.7) | 0.097 |
| Current alcohol user | 1.0 (0.8 - 1.3) | 0.966 |
| ***Socioeconomic*** |  |  |
| Distance from clinic (km; median (IQR)) | - | 0.180 |
| **Private medical insurance** | 0.5 (0.2 - 1.0) | **0.021** |
| **Unemployed** | 1.7 (1.4 - 2.2) | **<0.001** |
| Employment physical demands |  |  |
| Mostly sitting or standing | - | - |
| Mostly walking but not heavy lifting | 1.1 (0.7 - 1.8) | 0.601 |
| Heavy manual labor | 1.4 (0.7 - 2.6) | 0.360 |
| ***Injury*** |  |  |
| **Occurred during rainy seasons (Mar-May, Nov-Dec)** | 1.3 (1.0 - 1.6) | **0.035** |
| Mechanism of Injury |  |  |
| Pedestrian struck by vehicle | - | - |
| Motor vehicle crash | 1.1 (0.8 - 1.6) | 0.545 |
| Motor cycle crash | 1.1 (0.8 - 1.6) | 0.568 |
| Fall | 1.0 (0.6 - 1.7) | 0.934 |
| OTA classification |  |  |
| Type A | - | - |
| Type B | 1.0 (0.8 - 1.3) | 0.915 |
| Type C | 0.7 (0.5 - 1.1) | 0.133 |
| Multiple extremity injury | 1.4 (1.0 - 2.0) | 0.088 |
| ***Treatment*** |  |  |
| **Definitive fixation** |  |  |
| External fixation | - | - |
| Plate | 0.7 (0.4 - 1.9) | - |
| SIGN intramedullary femur nail | 0.9 (0.7 - 1.3) | 0.639 |
| Non-SIGN intramedullary femur nail | 0.7 (0.1 - 3.8) | 0.675 |

SD: standard deviation; km: kilometers; IQR: interquartile range; OTA: Orthopaedic Trauma Association; SIGN: Surgical Implant Generation Network

Table S2. Mapping survey questions to follow-up barrier domains.

| **Figure 2 domain** | **Femur study** | **Tibia study** |
| --- | --- | --- |
| Travel distance | Long travel distance | The hospital is too far away;  I moved to another region, so it was difficult to make a long journey for the follow up |
| Travel cost | Cost of returning for appointment | I cannot pay for transportation to reach the hospital |
| Fear of hospital payments | Fear of having to pay hospital bill | I might have to pay for the clinic visit; I might have to pay debts I owe to the hospital |
| Feeling well | Feeling well, no symptoms | I feel good/no symptoms and have no need to come back |
| Work obligation | Unable to get time away from work | I cannot take time away from work |
| Other medical issue | Other medical problem | Another medical problem prevents me from coming |

# Femur Study: Telephone loss-to-follow-up survey

Participant ID _____________________

Date of phone contact _____________________

Please ask the patient if they missed their follow -up appointments for any of the following reasons. Select all that apply:

 Long travel distance

 Cost of returning for appointment

 Fear of having to pay hospital bill

 Feeling well, no symptoms

 Given incorrect appointment time

 Incorrect contact information

 Missing contact information

 Following up with different doctor

 Unable to get time away from work

 Other medical problem

 Death

# Tibia Study: Telephone loss-to-follow-up survey

Participant ID _____________________

Please explore through open-ended questioning each reason below as to why the patient missed their follow -up appointments.

Check all that apply for why this patient did not attend clinic.

 I did not know that a 1-year follow-up visit was scheduled

 The hospital is too far away

 I cannot take time away from work

 I cannot pay for transportation to reach the hospital

 I moved to another region, so it was difficult to make a long journey for the follow up

 I might have to pay for the clinic visit

 I might have to pay debts I owe to the hospital

 I feel good/no symptoms and have no need to come back

 Another medical problem prevents me from coming

 The surgery and treatment did not help me

 I was not satisfied with the services provided at the hospital
